# Advancing knowledge translation practices to accelerate change in adolescent and youth sexual and reproductive health practice: a scoping review

**DOI:** 10.1186/s12961-026-01481-6

**Published:** 2026-04-21

**Authors:** Abednego Musau, Lindsey Reynolds, Nabeel Petersen, Matthew Wilson, Mary Phillips, Meghan Cutherell

**Affiliations:** 1 Population Services International, 28 Whitefield Place, School Lane, Westlands, PO Box 14355-00800, Nairobi, Kenya; 2 Pivot Collective, Cape Town, South Africa; 3 Population Services International, Washington, DC United States of America; 4 University Medical Centre Groningen, Groningen, Netherlands

**Keywords:** Knowledge translation, Adolescent sexual and reproductive health, Sub-Saharan Africa

## Abstract

More effective knowledge translation (KT) in the field of adolescent sexual and reproductive health (ASRH) could improve the speed at which effective health and behavioural interventions are delivered to young people, improving their overall health and well-being. Given the limited literature on KT for ASRH, this scoping review synthesizes the evidence on the barriers and facilitators to knowledge translation in ASRH in low- and middle-income countries (LMICs). Our review combines a rapid scoping review of relevant published and grey literature on the facilitators and barriers to knowledge translation of ASRH in LMICs, where available, and knowledge translation in health care settings and health policy making more generally, as well as key informant interviews (KIIs) with ASRH implementers, policy-makers and researchers on the factors that shape the translation of evidence to ASRH policy and practice in Africa. Two reviewers systematically used a predetermined search string to retrieve studies in eight databases. After applying exclusion criteria, 53 full-length studies were included. Researchers extracted data on the key areas of interest, including KT approaches, barriers, determinants, facilitators, key lessons and best practices. KIIs were conducted with 22 participants. Interviews were transcribed, summarized for themes and then analysed. Data were then reorganized into categories and key themes organized using the KT framework developed by Jacobson et al. Barriers and facilitators for KT in ASRH mirror those the health sector faces more generally, such as the strength of existing knowledge management practices, the relationship between knowledge producers and consumers, and differences in the needs of knowledge producers and knowledge users. Specific considerations for ASRH identified were an overemphasis on research products and an underemphasis on relationship building, the political nature of adolescent sexuality and the need to engage more diverse voices in knowledge production and translation.

**Supplementary Information:**

The online version contains supplementary material available at 10.1186/s12961-026-01481-6.

## Background

Despite improvements over the last 30 years, low-income and middle-income countries (LMICs) continue to bear the highest burden of diseases globally, and as a result, their populations carry an inequitable share of the world’s mortality and morbidity [1]. Health systems in LMICs face multiple challenges to strengthening service delivery and improving the quality of care. Access to and effective use of health research is paramount to addressing these challenges, improving health outcomes and reducing health inequalities [2]. Health care innovations are continuously being developed, but new products, services, and standards of care are slow to be adopted and diffused [3]. Given the resource shortages faced by health systems in LMICs, implementers and policy-makers are under pressure to use this evidence to roll out more effective solutions [4]. However, the use of evidence to improve policy and practice is far from straightforward.

Take the case of adolescent sexual and reproductive health (ASRH). A key challenge for adolescents in many contexts is the lack of appropriate, affordable, and acceptable sexual and reproductive health services [5, 6]. Insufficient availability and accessibility of ASRH services can have significant health and social consequences for adolescents. In developing regions, approximately 12 million girls aged 15–19 years give birth every year [7]. Complications during pregnancy and childbirth are currently the leading cause of death for 15–19-year-old girls globally, and babies born to young mothers face higher risks of low birth weight, preterm delivery and severe neonatal conditions [8]. Pregnancy and childbirth also profoundly affect young women’s educational outcomes and their income-earning potential, thereby shaping their life prospects, and those of their children [9]. The ASRH field has produced significant evidence regarding effective strategies to address gaps in availability and accessibility of services and to reduce the burden of unintended pregnancies and pregnancy-related complications [10]. While this has resulted in some improvements in reproductive health outcomes for adolescents, significant issues persist including limited reach or duration, continued implementation of interventions that have been shown to be ineffective and poor implementation of methods that have been proven effective [11]. The efforts of the ASRH field illustrate that even when there is clear evidence on the most effective approach, it does not guarantee health systems will be able to adopt evidence-based practices for improved health impact.

To improve the translation of evidence to policy and practice, researchers and health system actors have developed diverse strategies to put knowledge into action and expedite evidence-based change in practice or policy-making [12]. Knowledge translation, as defined by the Canadian Institute of Health Research, and adopted by the World Health Organization and others is:

*“A dynamic and iterative process that includes synthesis, dissemination, exchange, and ethically-sound application of knowledge to improve health […], provide more effective health services and products, and strengthen the health care system. This process takes place within a complex system of interactions between researchers and users.”*

This definition emphasizes that knowledge cannot simply be transferred from one place or person to another. Rather, it must be tailored and transformed to meet the diverse needs and preferences of different categories of knowledge users. The concept of knowledge translation describes a process in which evidence generated from research is used to inform decisions and change action [13]. Knowledge translation is complex and context dependent, and is shaped by resource allocation, infrastructure and interest groups, as well as by social factors [14].

The theory of knowledge translation developed by Jacobson et al. in 2003 argues that effective knowledge translation requires an in-depth understanding of the context [15]. The authors offer a framework to help knowledge translators increase their familiarity with intended user groups before embarking on knowledge translation activities. The framework consists of five domains: the “user group and its characteristics”, “issue under consideration”, “available research”, “research–user relationship” and “dissemination strategies”. This framework considers a diverse range of factors and their interactions influencing evidence incorporation into decision-making while providing a practical approach to analysing findings. Rather than assuming that knowledge translation (KT) is a unidirectional flow of information from knowledge producers to knowledge users, it acknowledges the importance of contextual and political factors. The framework posits that successful incorporation of evidence into policy-making requires the coming together of several domains and an in-depth understanding of how those domains influence one another. Although originally developed for use in high-income countries, Jacobson’s framework has been shown to be relevant in low-resource settings [14]. While much of the scholarship in knowledge translation has been in high-income countries, there is an increasing body of literature on how to facilitate this process in low- and middle- income countries (LMICs) [16, 17]. Recent systematic reviews have explored the barriers and facilitators of health-related knowledge translation in LMICs [13, 18]. This work finds that barriers to effective knowledge translation include low capacity, time constraints and limited financial resources. High-quality evidence may be hard to access and is often not appropriately contextualized, despite increasing recognition that contextualization of evidence is a facilitator of knowledge translation. A review of researcher and research institution capacity for knowledge translation reflected many of these same challenges and opportunities, while also noting the complexity of the policy-making environment in LMICs and the importance of relationships between communities of policy-makers and researchers [17]. In LMICs, much of the literature has focused on knowledge translation for health in general, despite the repeated calls for more targeted strategies to address specific issues in national or subnational contexts [17, 19, 20]. There is very little evidence available on the factors effecting knowledge translation for sexual and reproductive health, particularly for LMICs.

In this review, we sought to understand the barriers and facilitators related to translation of novel ASRH knowledge, where urgent action is needed if young people’s sexual and reproductive rights are to be realized. This review folds together two methodological approaches: a rapid scoping review of relevant published and grey literature on the facilitators and barriers to knowledge translation in LMICs, with an emphasis on sexual reproductive health (SRH) and key informant interviews with ASRH implementers, policy-makers, and researchers to explore their experiences and perspectives on the factors that shape the translation of evidence to ASRH policy and practice in Africa.

## Materials and methods

This study was conducted within the context of the Adolescents 360 (A360) project, which aims to increase demand and access to and uptake of voluntary modern contraception among adolescent girls (15–19 years) in Ethiopia, Nigeria, Tanzania and Kenya.

### Scoping review

The review was conducted in accordance with the guidance on scoping reviews from the Joanna Briggs Institute (JBI) and using modified Preferred Reporting Items for Systematic Reviews and Meta-Analyses Extension for Scoping Reviews (PRISMA-ScR) [21]. The completed PRISMA-ScR checklist is included (Supplementary File B). The scoping review methodology was selected for its flexibility and aim to provide a descriptive overview of reviewed material, which is well suited to an analysis of barriers and facilitators. As our initial exploratory review revealed limited peer reviewed literature about ASRH knowledge translation in LMICs, we included other sources, including reports and webpages of key organizations in this field.

#### Search strategy

The search strategy was guided by a detailed protocol, which is unregistered but available on request. To begin developing a database search strategy, the research team reviewed key words in the objective of the review. The following concepts formed the base of the search strategy “knowledge translation”, “health service provision”, “adolescents”, “low- and middle-income countries” and “sexual and reproductive health”. Additional possible synonyms were generated and refined through an initial exploratory review and web searches, and then were mapped, sorted, and refined by the full research team. These were incorporated in the key search term through BOOLEAN operators to identify relevant studies (Table 1).

**Table 1:** Scoping review search terms

Key word	Search key	Search terms
Knowledge translation	S1	“Knowledge translation” OR “translation knowledge” OR “Translational medical research” OR “implementation science” “research uptake” OR “implementation research” OR “knowledge transfer” OR “research utilisation” OR "translation of research” OR “translating evidence” OR “Evidence based” OR “use of evidence” OR “research based” OR “evidence transfer” OR “research uptake” OR “research informed” OR “evidence dissemination”
Health service provision	S2	“Health service provision” OR “implementation” OR “health personnel” OR “health strategy” OR “health policy” OR “decision making” OR “public health policy” OR “policymaking” OR “policy making” OR “health services research” OR “health administrators”
Adolescents	S3	“adolescents” OR “young people” OR “teenagers” OR “youth” OR “young person” OR “adolescent health”
Low- and middle-income countries	S4	“Low-and middle-income countries” OR “sub-Saharan Africa” OR “Africa” OR “global south”
Sexual and reproductive health	S5	“Sexual and reproductive health” OR “sex education” OR “sexual health” OR “reproductive health” OR “contraception” OR “family planning services” OR “condom use”

Two independent reviewers (LR and NP) systematically used the search string to explore and retrieve studies in PubMed, EBSCOhost, Cumulative Index of Nursing and Allied Health Literature (CINAHL), Educational Resource Information Center (ERIC), Medical Literature Analysis Retrieval System Online (MEDLINE), Academic Search Premier and Sabinet as well as Google Scholar. In the PubMed search, MeSH terms were applied across all key terms to ensure that all relevant articles were extracted for review. These database searches were supplemented by focused data searches via the internet to gather information related to the topic from websites and grey literature sources.

#### Inclusion and exclusion criteria

We began the search strategy with stringent criteria to identify studies that focused on (i) ASRH knowledge translation barriers, (ii) ASRH determinants of knowledge translation, (iii) best practices and (iv) recommendations for ASRH knowledge translation. However, because of the paucity of evidence of knowledge translation in ASRH in LMICs the inclusion criteria were subsequently relaxed. We therefore redirected and broadened the inclusion criteria to encompass knowledge translation in health care settings and health policy-making in general.

We limited our sample to studies conducted in low-income or LMIC settings. The review was conducted between September and December 2021, and we reviewed studies published in English between 2010 and 30 August 2021. This timeframe enabled us to focus on the existing state of the field of knowledge translation in adolescent sexual and reproductive health at the time of the review. Articles were sorted and coded based on publication type (peer reviewed published articles, websites, reports, commentary).

#### Article screening

All articles generated from search engines were exported to the Zotero reference manager. Three reviewers (LR, NP and a research assistant) identified potentially eligible studies using the criteria and independently screened titles and abstracts for relevancy. Any discrepancies or doubts between the primary reviewers were resolved by the senior researcher (LR). Following the initial screening, selected sources were retrieved for full-text screening. Each reviewer then independently reviewed a set of full-text articles. Data from each article were abstracted and inputted into a standardized form. The screening process was done iteratively, with team discussions held weekly to establish consensus on provisionally included articles. Citation analysis of the selected sources revealed an additional 100 potential articles for inclusion which were then reviewed by the research team, 4 of which were duplicates and 77 which were excluded.

#### Data extraction and synthesis

To ensure data were consistently extracted from source material, the reviewers developed a form with oversight from the senior researcher (Supplementary File A). Extracted data included the primary author’s name, study setting, aim/question, study population, study design, sampling method, sample size, knowledge translation approach/intervention barriers to knowledge translation, knowledge translation determinants, knowledge translation facilitators, key lessons and best practices. Each paper was also coded using pre-determined codes. These were kept in line with the objective specifically focusing on the (a) key features – inclusion criteria, (b) intended effects – the outcomes linked to intervention or knowledge translation strategies and, (c) key lessons.

Results were organized thematically by adopting a blended approach to analysis that encompassed both deductive and inductive frameworks. Data were reorganized into categories and key themes. From the review, we then identified facilitators and barriers to knowledge translation in health care as well as the key programmes/interventions used to drive knowledge translation and related outcomes. A constant comparison approach across studies was used to document findings. Meta-narrative allowed for acknowledgement of diverse forms of evidence to understand complex issues while identifying key features of knowledge translation. We use Jacobson et al. [15] as a framework to organize what we learned during our review.

### Key informant interviews

Given the limitations in the existing evidence base, we found that the scoping review was insufficient to meaningfully address the central mandate of the review. As a result, the study team designed a second phase of data collection focused on gathering insights from key stakeholders. Key informant interviews (KIIs) were conducted with three groups of respondents in each of the four project countries: (1) ASRH policy-makers and implementers, (2) staff from the A360 programme and (3) ASRH researchers. These individuals were based in the programme’s implementation countries: Kenya, Ethiopia, Nigeria and Tanzania. In addition, based on lessons from the first round of interviews about important influencers in ASRH in the region, the team recruited and interviewed a group of regional experts who could reflect on the study questions from a broader perspective.

Interviews covered the following themes: (1) motivations and commitments in ASRH work; (2) networks, relationships, trust and credibility in ASRH knowledge flows; (3) how and by whom ASRH knowledge is produced; (4) how and by whom ASRH knowledge is consumed; (5) engagements with evidence; (6) facilitators and barriers to knowledge translation in ASRH; (7) strategies to ensure knowledge is actionable; (8) packaging of knowledge/evidence; and (9) knowledge needs/wants. Interviews were audio recorded with participants’ consent. The research team transcribed interviews verbatim and then developed anonymized case summaries. The team then conducted a rapid thematic analysis of the case summaries to identify key insights and cross-cutting themes following the guidance by Braun and Clarke [22].

## Results

**Fig. 1:**
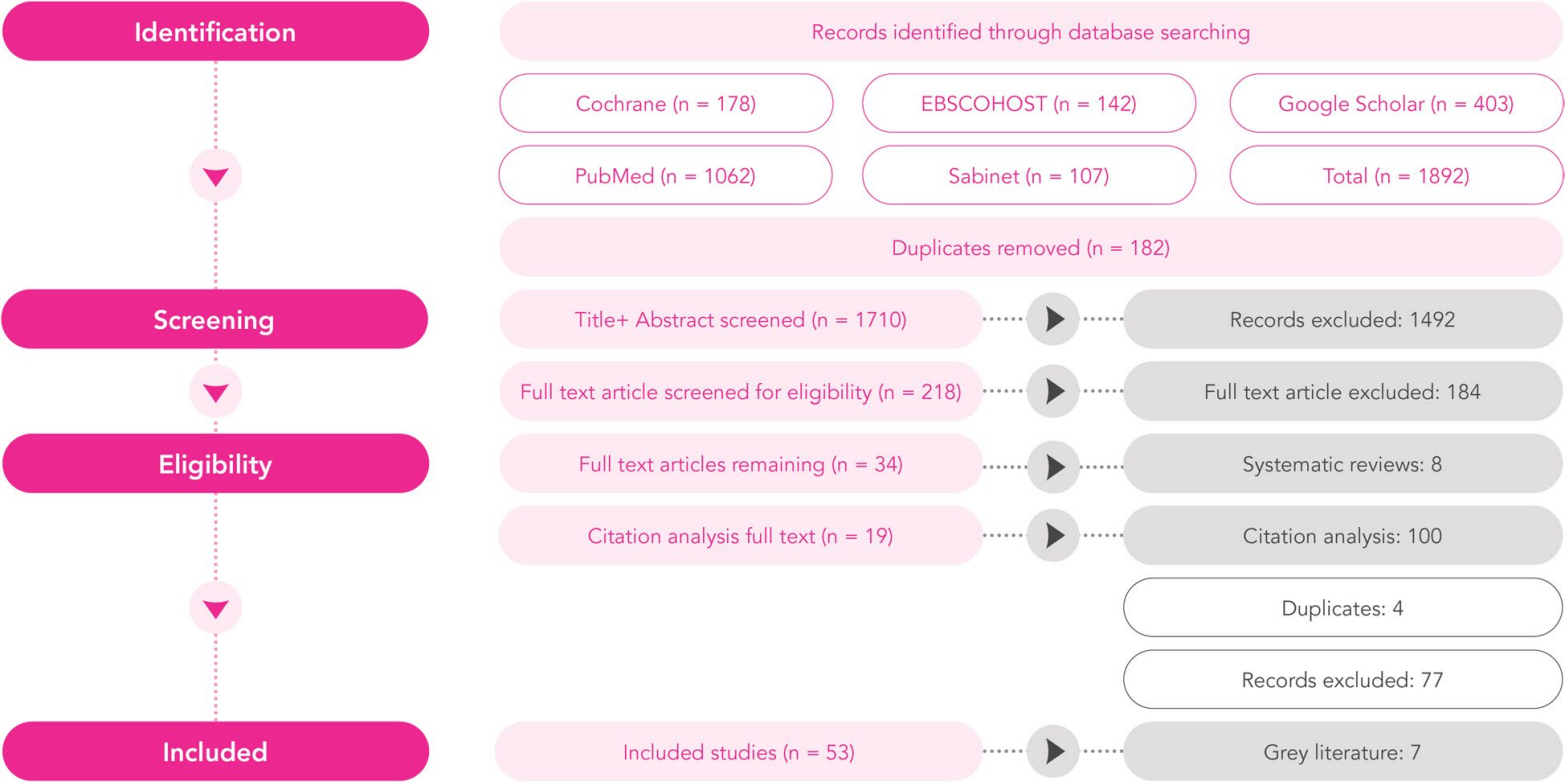
PRISMA flow diagram

In total, 1892 articles were retrieved from the following databases: PubMed (*n* = 1062), EBSCOhost (*n* = 142), Cochrane (*n* = 178), Sabinet (*n* = 107), Google Scholar (*n* = 403). After duplicate articles were removed, 1710 articles remained. Following the screening of article titles and abstracts for inclusion and exclusion criteria, 218 articles were selected for full-text review. Of these, 34 were deemed eligible. Based on the citation analysis a further 19 full text articles were retrieved and reviewed. Ultimately, 53 articles were selected for detailed analysis. See the Prisma Flow Diagram in Fig. 1 for the details of the screening process.

Of the sources included, 64% (*n* = 34) were multinational, comprising studies conducted across two or more African countries or other LMICs. African countries represented included Burkina Faso, Cameroon, Central African Republic, Ethiopia, Ghana, Kenya, Malawi, Nigeria, Senegal, South Africa, Sudan, Uganda and Zambia. Other LMICs represented included Argentina, Bangladesh, Bolivia, Brazil, Cambodia, Colombia, Dominican Republic, Indonesia, Iran, Laos, Malaysia, Mexico, Myanmar, Nepal, Nicaragua, Peru, Philippines, Sri Lanka, Thailand and Vietnam. Of the remaining studies conducted in one country, the most common countries were Ethiopia (*n* = 3), Ghana (*n* = 2), Iran (*n* = 2), Kenya (*n* = 3), Nigeria, (*n* = 2), Senegal (*n* = 2), South Africa (*n* = 2), Uganda (*n* = 3) and Zambia (*n* = 2). Included studies used different research designs, although most of the studies were qualitative in nature. Methodologies included interviews (*n* = 16), surveys (*n* = 1), case studies (*n* = 13), cross-sectional studies (*n* = 5), mixed-method studies (*n* = 3), panel discussions (*n* = 1) and evidence mapping (*n* = 1).

A total of 22 interviews were conducted over the course of 3 months. As depicted in Table 2, 14 women and 8 men were interviewed. The geographic breakdown was as follows: Kenya (*n* = 4), Ethiopia (*n* = 3), Nigeria (*n* = 6), Tanzania (*n* = 3) and regional experts (*n* = 6). The majority of respondents self-identified as having multiple roles simultaneously in the knowledge production and translation process, including producers, consumers, implementers and translators.

**Table 2:** Interview participant characteristics

	Number	%
Programmatic country of focus		
Kenya	4	18
Ethiopia	3	14
Nigeria	6	27
Tanzania	3	14
Regional expert	6	27
Sex		
Male	8	36
Female	14	64
Years of experience in knowledge translation		
< 10	1	5
10–20	5	23
> 20	5	23
Unknown	11	50
Role in knowledge production process*		
Knowledge producer	14	64
Knowledge consumer	13	59
Knowledge translator	6	27

^*^Multiple responses possible

### Reported findings

The review of the published literature revealed a variety of documented barriers, facilitators and strategies for strengthening KT in LMIC settings. Informants echoed many of these findings, such as the challenges related to existing knowledge management practices, relational barriers between knowledge producers and consumers, and structural differences in the knowledge production versus knowledge use (policy and practice) space. Table 3 maps these considerations with illustrative quotations from the KIIs, to Jacobson et al.’s framework. Given the specific attention given to KT in ASRH in our review, “the issue” dimension of the framework is not included in the table but is discussed in greater detail afterwards.

**Table 3:** Findings mapped to user context domains

Dimension	Key findings	Illustrative quotation from interviews	Articles
User group	(−) Lack of time or inclination to read	“Really, it’s just my busy schedule, I don’t think there are any other barriers.” (T1)	Crichton and Theobald [23]; Oronje et al. [36]; Sylla et al. [31]; Khammarnia et al. [27]; Guieu et al. [25]; Jessani et al. [26]; Kirigia et al. 2016 [28]; Kalibala and Nutley [19]; Langlois et al. [29]; Pather and Mash [30]; Murunga et al. [17]; Dagne and Tebeje [24]; Kalbarczyk et al. [16]
	(−) Digital inequalities	“The biggest challenge is the internet. The information is there if you search, but you may not have the right platform or the network might be challenging… The infrastructure, equipment, software to do it needs to be correct.” (N2)	Sylla et al. [31]; Khammarnia et al. [27]; Edwards et al. [18]; Dagne and Tebeje [24]
	(−) Gaps in the skills needed to appraise, understand and apply research	“When you share knowledge, consumers don’t have the skills to use your products. We should offer more technical support to help consumers implement. You can’t just hand over evidence.” (N6)	Crichton and Theobald [23]; Oronje et al. [36]; Sylla et al. [31]; Nabyonga Orem et al. [34]; Hardee et al. [32]; Khammarnia et al. [27]; Jessani et al. [26]; Uzochukwu et al. [39]; Vogel et al. [38]; Ogbe et al. [35]; Kalibala and Nutley [19]; Islam et al. [33]; Murunga et al. [17]; Dagne and Tebeje [24]; García-Cerde et al. [46]; Shilton et al. [37]
	(−) Low perceived benefit or value of knowledge products	“Another issue has to do with the perceived benefit/value proposition of the knowledge products. Consumers need to see the benefits of using the knowledge products.” (N6)	Khammarnia et al. [27]; Uzochukwu et al. [39]; Dagne and Tebeje [24]
The research	(−) Lack of high-quality, contextually relevant knowledge	“The government data is a little difficult to trust all the way because of data quality issues, like the epidemiological data. Even they themselves hardly use their own data because there are so many issues around the quality. Mostly like small CBOs, who are doing their own work in this area, who may be producing materials which is focused on small projects. We may use it to highlight a case study or programme, but not drawing wholly from it as it won’t be representative. It might have quality issues that we cannot speak to and it could affect the findings or whatever is being reported.” (K4)	Kirigia et al. [28]; Kalibala and Nutley [19]; Oronje et al. [20]; Murunga et al. [17]
	(−) Lack of costing data	“The fear that the evidence that is generated may be too expensive to implement is a major barrier. Lack of costing data is a key issue.” (N6)	Kirigia et al. [28]; Shilton et al. [37]
Researcher–user group relationship	(−) Disconnect and/or lack of trust between knowledge producers and users	“First, policymakers and academics have some basic mistrust of each other. Sometimes the language academics speak does not cause policymakers to trust them. They take it as a sort of lack of reality. They don’t speak the same language. Policymakers just want to digest the knowledge. Second, they have different worldviews. For many researchers, their ultimate aim is publishing one high rated article in one big peer reviewed journal. Sometimes as beautiful as these are, they are not actually addressing what matters and what policymakers care about. Many times we are talking about people who are in the same field, but they don’t actually cross paths very much. There is very little intention to infuse ideas, share knowledge, and learn from each other.” (N1)	Nabyonga Orem et al. [34]; Jessani et al. [26]; Kirigia et al. 2016 [28]; Uzochukwu et al. [39]; Asaku [40]; Kalibala and Nutley [19]; Islam et al. [33]
	(−) Unequal power dynamics while creating and using evidence	“Another big issue is that we sometimes, perhaps systematically, exclude the voices of those in the most marginalized communities. We don’t get to hear the other people who are the ones actually affected and seeing what’s happening. People who are finding evidence at local levels often don’t have funding/support and it takes a lot to get the evidence generated. When you compare meetings at national level, they don’t get the space to hear their opinion or let their work speak to the larger national platform. We systematically exclude those kinds of community-based information. In the development world we hear the loudest voices with the greatest privilege, and thus we don’t get to hear those at community level. That critical evidence can be most important to changing things at community level. This is because of the way the research world is constructed and the way we constructed the research–policy interaction.” (N1)	Sylla et al. [31]; Kirigia et al. [28]; Uzochukwu et al. [39]
	(−) Misaligned timeframes	“A researcher wants to do a deep study, get the best of data and a policymaker has to respond to issues very quickly. The average policymaker thinks in a 2-year cycle in terms of delivering policy – one year to get established, second year to do things, and then the next year to prepare for their next campaign. Researchers don’t work in a fast enough time frame. By the time the researcher produces a result there is very little time for use.” (N1)	Nabyonga Orem et al. [34]; Hardee et al. [32]; Guieu et al. [25]; Kirigia et al. [28]; Oronje et al. [20]; Pather et al. [30]; Islam et al. [33]
	( +) Ongoing stakeholder involvement in research design and program implementation	“Where policymakers were actively involved throughout the process – not at the just at the end of the project – usually those are the ones that are trusted. Because they were involved through all the learnings. Whether they were failures or successes – this is the knowledge that gets taken up. At the policy desk, the policy maker wants to be involved throughout the process, understanding the successes and failures so that they can internalize whatever learnings. And when you do have the final synthesis, they see themselves as involved/as part of it. If they have contributed and engaged along the way, they see themselves as co-owners.” (N3)	Oronje et al. [36]; Ssengooba et al. [47]; Tulloch et al. [44]; El-Jardali et al. [48]; Shroff et al. [14]; Kok et al. [49]; Uzochukwu et al. [39]; Ogbe et al. [35]; Varallyay et al. [50]
	( +) Strong relationships between evidence producers and consumers	“If you give participants the opportunity/ freedom to share their inputs/views in this space, then useful collaboration can come out of it, to shape and improve our implementation. That brings the sense of ownership, it not just government led, its government and the partners. The ownership brings a sense of collaboration.” (T1)	Oronje et al. [36]; Ssengooba et al. [47]; Theobald et al. [43]; Tulloch et al. [44]; El-Jardali et al. [48]; Ongolo-Zogo et al. [42]; Guieu et al. [25]; Jessani et al. [26]; Langlois et al. [29, 51]; Uzochukwu et al. [39]; Vogel et al. [38]; Ogbe et al. [35]; Varallyay et al. [50]; García-Cerde et al. [46]
	( +) Provision of technical support to policy-makers to use knowledge products	“Ongoing technical support provision is key to helping countries move from ‘intention to act’ to ‘action’." (R5)	Kirigia et al. [28]; Langlois et al. [51]; Oronje et al. [20]
	(−) Lack of institutionalized and systematic knowledge exchange platforms	“Kenya lacks a space or repository for emergent evidence… In the case of a policy document that was formulated some years back, for instance, there is no way you could find that document because it was not uploaded online.” (K4)	Nabyonga Orem et al. [34]; Kirigia et al. [28]; Malla et al. [13]; Kalibala and Nutley [19]
Dissemination strategies	( +) Frame and package knowledge products for specific audiences	“The key to effective knowledge translation is knowing that you don’t just share the data. You need to help policymakers make sense of it. This involves finding the right way of framing the data. For instance, you need to show the effects of issues related to SRH. For instance, to get policymakers concerned about low contraceptive use, you must focus on the problem of maternal mortality.” (N6)	Hyder et al. [41]; Theobald et al. [43]; Tulloch et al. [44]; Nabyonga Orem et al. [34]; Ongolo-Zogo et al. [42]; Shroff et al. [14]; Jessani et al. [26]; Uzochukwu et al. [39]; Oronjeet al. [20]
	( +) Knowledge products offer clear policy recommendations	“You have to tell people what you want in very plain language and very actionable steps. Because they don't have time to think about how to break down the evidence that you are presenting into ‘what are you asking of me’ – if someone has to ask that question then you already not doing a great job at explaining the information. (R4)	Hyder et al. [41]; Oronje et al. [36]; Theobald et al. [43]; Tulloch et al. [44]; Mash et al. [45]; Kalibala and Nutley [19]
	( +) Increased range of types of knowledge products	“I don’t think one size would fit all. I love communities of practice because they bring different actors together using webinars, bulletins, etc. I also think we should encourage data story books. I also really like infographics as ways to share key insights. Experience sharing is also great through more active knowledge exchange. Also using new digital media really allows for young people themselves to engage in the knowledge process. Participatory information and knowledge sharing is very important in digital media spaces.” (N4)	Islam et al. [33]; Murunga et al. [17]
	( +) Promote knowledge sharing and knowledge use	“Giving policymakers and academics the opportunity to sit together and learn/share from each other. We need more platforms for us to work together. If researchers begin to talk to policymakers and use their problems to structure their research questions, that would really help.” (N1)	Ssengooba et al. [47]; Oliver et al. [52]; Ongolo-Zogo et al. [42]; Uzochukwu et al. [39]; Malla et al. [13]; Edwards et al. [18]; Islam et al. [33]

### Challenges in knowledge access, quality and utility

Our review confirmed that the knowledge users face several barriers to identifying and using knowledge in their work. These barriers include limited time with which to engage new information, especially when that information is presented in written form [17, 19, 20, 23–31]. They are likely to lack access to new research, because of poor internet connectivity or restrictive paywalls [18, 24, 27, 31]. There was an acknowledgement of a critical skills gap in analysing and using data, which meant that information shared passively was unlikely to be used. Often, the user group was seen to undervalue knowledge products and be unclear on how the products could be used to strengthen their ongoing work. Within the research dimension, there were consistent concerns about the quality and applicability of data [17, 19, 23, 26, 27, 31–39]. A tension exists between the data perceived as “high-quality” or derived from rigorous studies, which is often conducted in other contexts or fails to address key outcomes of local interest and the data that is local and relevant but considered “lower quality” [17, 19, 20, 28]. This results in uncertainty among knowledge users about how to use data to make decisions. In the KIIs, in particular, there was skepticism about the value of data provided by nongovernmental organizations (NGOs), who may be incentivized to present more flattering pictures of their own work to retain donor funding. An additional concern is the lack of costing data around interventions and approaches, which is especially frustrating to policy-makers [28, 37].

### Barriers and facilitators in the researcher–user relationship

The dimension of researcher user-group relationship revealed both barriers and facilitators to knowledge translation. Disconnection, lack of shared priorities, misaligned timelines, and power imbalances hurt this relationship and make it hard for knowledge to transfer effectively [19, 26, 28, 33, 34, 39, 40]. The review also confirmed the inadequacy of simple categorizations of “knowledge user” and “knowledge producer”. Most of our KII participants identified as both knowledge users and producers. We also heard from the respondents that we need to consider groups beyond researchers and policy-makers, to include NGOs, youth voices and community leaders. Within this widened understanding of the actors in knowledge production and translation, the question of power becomes more prominent, with respondents warning that those not in traditional positions of knowledge production (researchers) or knowledge use (policy-makers), may not be listened to, which further reduces the likelihood of relevant knowledge being produced or used locally [28, 31, 39]. On the other hand, this dimension offers many positive facilitators of knowledge translation if strong relationships can be cultivated through ongoing engagement, targeted relationship building and provision of technical support. A practical concern raised in this dimension was the need for knowledge exchange platforms that can be used by both producers and users.

### Audience-centred dissemination strategies

Within the domain of dissemination strategies, the review showed the need to be more thoughtful about users’ preferences when producing knowledge products, particularly by using accessible language and ensuring research findings are not restricted by paywalls [14, 20, 26, 34, 39, 41–44]. There was a sense that products should be more carefully packaged for specific audiences and, for those directed at policy-makers, include clear policy recommendations [19, 36, 41, 43–45]. There was a stated desire to see a broader range of knowledge products, beyond peer-reviewed journal articles and to communicate more creatively using channels users were already engaged in, such as social media [17, 33].

Across the interviews, three additional themes emerged as critical considerations for KT in AYSRH: (1) overemphasis on research products and underemphasis on relationship building, (2) the political nature of adolescent sexuality and (3) youth engagement for contextualizing knowledge.

#### Overemphasis on research products and underemphasis on relationship building

Although the need for targeted dissemination strategies and the use of multiple types of knowledge products is well documented [17, 33], the respondents went a step further by indicating that there was an over-reliance on more formal and technical knowledge products, which were more difficult to access and understand and harder to make actionable. It was notable that respondents indicated not just preference for more collaborative, targeted forms of knowledge sharing products, but often active dislike or frustration with more traditional, academic research products, such as peer-reviewed journals.

*“Why should people pay or subscribe to know what is happening? One of the main reasons people do research is to leave a mark or advocate for change, so if you are limiting people getting that knowledge and getting that evidence, it makes no sense.”* (K4)

*“Producing knowledge in everyday language and in simple formats that the average policymaker and programmer can digest and utilize. [Peer-reviewed journals] of the world are too dense for this use.”* (N1)

The need for increased collaboration and trust between researchers and policy-makers is also well documented [25, 26, 29, 35, 36, 39, 42–44, 46–48]. However, respondents emphasized that the knowledge products that are most likely to be effective are the ones rooted in the researcher–user relationship. While Jacobson et al.’s framework considers the user relationship and the dissemination strategies as separate domains, respondents often spoke about them as deeply intertwined and were less interested in any products that were not developed through this partnership.

*“The packaging of the knowledge should be co-designed with the intended audience for relevance and assurance that the message is received by those it is intended for. For example, a 30-page document directed toward a young person or a busy policy decision-maker will not work.”* (E1)

*“Most of the time, the most effective strategies are through technical working groups and workshops. Walking government through to show exactly how it is done. It’s a good thing to do posters and banners and peer reviewed literature but to get policy into practice in Nigeria, it’s all about the relationships.”* (N5)

#### Political nature of adolescent sexuality

When examining the responses related to Jacobson’s second domain, “the issue”, respondents reflected that in the context of ASRH another important barrier to effective knowledge translation is the discomfort of many policy-makers, health service providers, and other key actors with discussions of sex and sexuality, especially when these involve adolescents. This discomfort results in restrictive policy contexts, which actively impede the translation of ASRH evidence into practice. Respondents reflected on how this discomfort can manifest both in overly restrictive policy that severely limit adolescents’ sexual and reproductive rights as well as, or alternatively, with an unwillingness to engage with the topic.

*“Another challenge is in relation to national regulations and laws. In some countries, there are still regulations and laws that do not protect adolescents’ and young people’s rights.”* (R2)

*“The stakeholders in those places, which are contested areas, the religious groups, traditional leaders, politicians, policy makers, they are not comfortable with speaking around issues of SRH of young people so that is a serious limitation.*” (K3)

While taboos on adolescent sexuality vary based on context, this discomfort is widespread and those working to translate knowledge in this arena must account for the sensitivity of this particular topic. Advocates for AYSRH may be familiar with this barrier in the context of programme implementation, but researchers and other knowledge producers may be less prepared to overcome or address the politicized elements of AYSRH.

#### Engaging more diverse voices in knowledge production and translation

As noted above, both the literature and many respondents reflected on the limitations of the evidence available for local application. Either the knowledge available was not contextually appropriate, was of poor quality or did not respond to key policy concerns [24, 27, 39]. Respondents were eager to see the production of more high-quality, local data, citing work that is already being done to produce and apply evidence locally.

*“Because there is so much [evidence] being produced in Nigeria and it is so diverse (different sociodemographic types of young people), we tend to rely on data that comes out of Nigeria, or at least sub-Saharan Africa, and not so much on broader global research. This is especially true for qualitative data, where we tend to focus quite heavily on Nigerian sources.” (N5)*

*“Ensuring that research speaks to the reality of the challenges within the country or the communities. When researchers pay attention to this and consult with policymakers around the policy issues that they are struggling with and the questions they are looking for answers to, it will have more influence – knowledge will be more desirable.” (N1)*

Respondents were clear that for this to work, we must think beyond the traditional two-way system of knowledge translation where the only actors are researchers and policy-makers engaged in a push–pull relationship. Instead, it is essential to ensure that diverse voices are involved, including not only policy-makers but also public health experts, programme implementers, lecturers, representatives of academic institutions, young people representatives and civil society representation [14, 35, 36, 39, 44, 47–50]. “We need to receive the perspective of all those people in revising or developing new guidelines,” one regional policy-maker shared. “It must be an inclusive process” (R2). Another explained:

*“ASRH is broad, it needs to be seen from a beneficiary point of view, from a policy maker point of view, from a technical point of view, etc. There are so many different angles we need to do research on… I think leveraging on each other is a way of ensuring that we collect enough knowledge from different angles and perspectives to give us the complex solution to the complex problem.” (R3)*

In particular, for knowledge translation in ASRH, respondents urged the greater inclusion of youth voices. Young people can ground learning in their own lived experiences and tacit forms of knowledge, knowledge that is rooted in experience and intuitive know-how, rather than over-relying on “expert” knowledge.

*“What works is working with people who can contextualize, localize and internalize the knowledge themselves and not relying too much on ‘experts’ who speak about high-level evidence. We really need to pay more attention to local contexts. Again, humans are not homogenous. We need to bring in those who are from different settings.” (N4)*

*“Adolescents and young people have the capacity to engage should they be provided with platforms for inclusion as they are the experts at being young… Missing that expertise is something that is not allowing us to achieve what we want to achieve. They should be involved in each and every aspect of the work that we are doing… when young people act and get the right opportunity and the right support, they shine.”* (E4)

Respondents noted the need for additional work in this area, to support and encourage adolescents and young people to advocate for themselves. This may require providing them with the knowledge they need to become advocates for their own SRH rights.

*“We also see a number of NGOs starting to use social media to influence positive behaviour by providing young people with access to the correct information/knowledge through the same channels that they access. Adolescents are now coming in on their own and are becoming more trusting of health personnel, asking for services and specific kinds of support, and asking institutions who are doing similar programs.”* (N4)

## Discussion

This review confirms that many of the barriers and facilitators in ASRH mirror those the health sector faces more generally for knowledge translation. Challenges related to existing knowledge management practices, relational barriers between knowledge producers and consumers, and structural differences in the knowledge production versus knowledge use space continue to drive the research-to-practice gap. One challenge specific to the ASRH field is the political sensitivity of adolescent sexuality and the discomfort knowledge producers and users may have engaging on this topic, either to understand its drivers or consider its solutions. Our review deepens understanding of the importance of the knowledge-producer–knowledge-user relationship not just to be a precondition for knowledge translation, but to be a fundamental element of any successful dissemination strategy. Additionally, the review highlights the importance of diverse voices in knowledge production and translation. Our analysis of these elements for KT in ASRH against Jacobson’s et al.’s framework may support more rapid assessments for others looking to be more effective in their KT in this health area.

The sensitivity of ASRH is well documented. Topics such as contraception, abortion, menstrual hygiene, and intimate partner and sexual violence continue to preclude knowledge producers from designing research to evaluate the effectiveness of potential interventions, including digital platforms that could amplify universal access to health promotion and service utilization interventions [53]. The controversies surrounding these topics also strain the relationship between researchers, communities and policy-makers. The social, cultural and religious barriers around sexual activity, particularly before marriage, can lead to restrictive policy environments and stigma for adolescent girls who seek services, while negative gender norms that promote early marriage and childbirth can make promotion of reproductive health be perceived as a threat to long-held beliefs [54]. To overcome these barriers in the policy arena, suggested actions include framing research findings to align with existing beliefs or popular ideas and changing the way policy-makers view disadvantaged groups [55]. Those seeking to transfer knowledge in ASRH must be aware that the topic is often fraught and understand what challenges and opportunities around adolescent sexuality present in the local context. While the importance of addressing negative social norms for contraceptive uptake is well documented, less is known about how to manage these sensitivities to increase receptiveness for improved policy and practice at a system level [56]. Emerging evidence shows that fully involving adolescents and their influencers throughout the research cycle can produce needs-driven research and strengthen advocacy to reform restrictive policies [57].

The reflections on evidence quality and the call to engage more diverse voices in knowledge production and translation may be useful to those seeking to improve knowledge translation practices for health areas beyond ASRH in LMICs. The evolution of the global research priorities for adolescents’ sexual and reproductive health and rights was possible due to a multistakeholder process involving programme implementers, researchers, civil society, funders, policy-makers and young people [58]. Such examples should be adopted with adaptations to contextualize the engagement process in the knowledge translation process. Calls to localize evidence production and ensure that knowledge production does not reflect historical power imbalances have recently taken a step further through arguments for the need to reduce reliance on biomedical solutions alone and value evidence that is tacit, or based in experiential knowledge [59, 60]. While high-quality, rigorous studies will remain essential in the field of global health, there is increasing recognition that, for effective solutions to scale, more attention needs to be given to contextual factors [61, 62]. Inclusion of more local, varied voices to strengthen evidence production has the potential to be a more sustainable, equitable and effective way to approach knowledge translation. Importantly, efforts to widen knowledge translation should be accompanied by investments to expand the capabilities of disadvantaged stakeholder groups to meaningfully participate in generating and utilizing research while strengthening existing institutions for sustainability [13].

The review has some limitations. First, it focused only on work authored in English, which may have excluded relevant articles produced in other languages. Secondly, while search terms were selected carefully, the field of knowledge translation and ASRH are both vast and exclusion of a key term may mean we missed relevant evidence. Third, the delay between completing the review and producing this manuscript may mean that we have missed new evidence on the area of focus which could have added to the findings presented. The review should be interpreted with these limitations in mind.

## Conclusion

This review has highlighted multiple barriers and facilitators for knowledge translation for ASRH in sub-Saharan Africa. The barriers and facilitators identified by the review reflect common knowledge translation practices in most sub-Saharan Africa health systems, including weak knowledge management practices, the strained relationships between knowledge producers and users, and misaligned needs between knowledge producers and users. Strengthening knowledge translation for ASRH can be achieved by fostering stronger relationships between producers and users, acknowledging the political dimensions of adolescent sexuality, and actively engaging diverse voices to enrich research production and translation.

## Supplementary Information

Supplementary Material 1.

Supplementary Material 2.

## Data Availability

The review articles are available through various platforms. Transcripts derived from the interviews could be availed by the corresponding author through a reasonable request. These transcripts could not be sufficiently de-identified and are not publicly available, due to ethical considerations.
